# Exploring 4 years of HIV pre-exposure prophylaxis as a statutory health insurance benefit in Germany—a longitudinal claims data analysis

**DOI:** 10.3389/fpubh.2025.1586736

**Published:** 2025-11-05

**Authors:** Sandra Schmischke, Christian Kollan, Ursula Marschall, Daniel Schmidt

**Affiliations:** ^1^Department of Anesthesiology and Intensive Care Medicine, Charité –Universitätsmedizin Berlin, Corporate Member of Freie Universität Berlin and Humboldt-Universität zu Berlin, Berlin, Germany; ^2^Department of Infectious Disease Epidemiology, Robert Koch Institute, Berlin, Germany; ^3^Medicine and Health Services Research, BARMER, Wuppertal, Germany

**Keywords:** HIV pre-exposure prophylaxis (PrEP), Germany, statutory health insurance (SHI), claims data, proportion of days covered (PDC)

## Abstract

**Background:**

Introduction of HIV pre-exposure prophylaxis (PrEP) into German statutory health insurance in September 2019 reduced barriers to an effective tool for HIV prevention.

**Objective:**

This study aims to provide a longitudinal analysis of HIV-PrEP care in a real-world setting in Germany between 09/2019–08/2023 using claims data from the insurance provider BARMER.

**Methods:**

We assessed anonymized claims data to analyze PrEP care and user characteristics. PrEP courses were defined and analyzed based on initiations, discontinuations and reinitiations, as well as proportion of days covered (PDC) as metric of adherence, post-exposure prophylaxis (PEP) and incident HIV infections.

**Results:**

A total of 5,208 PrEP users with a median and average observation time of 1.3 and 1.7 years were identified, totaling to 8,822 person-years. By the end of observation 58% were active PrEP users and 43% were still on their first PrEP course. Median age was 34 years (IQR 28–43) and 98.6% were male. Median PDC was 91.3% (IQR 78.9–98.6%) for all observed courses. PrEP initiations increased again in 2022 after a decrease in 2020 and 2021. Of 267 PEP courses, 73.8% took place before PrEP initiation. HIV incidence rate was 0.07/100 person-years. Out of the six detected HIV infections five occurred after PrEP discontinuation.

**Conclusion:**

Overall PrEP pill coverage was high and PrEP proved to be highly effective in preventing HIV. No stagnation or decline in PrEP uptake was observed, suggesting that PrEP needs in Germany are not yet fully covered. In 4 years of observation we saw a considerable number of PrEP discontinuations and reinitiations, which indicates that PrEP is utilized in accordance with specific user needs. Most PEP occurred before PrEP initiation, indicating that HIV risk was recognized which eventually led to PrEP prescription. We found a negative association between individuals’ total PDC and length of PrEP use, suggesting need for interventions to increase adherence among long-term PrEP users. This study supports previous findings of high PrEP demand in men, assumingly mostly men who have sex with men and suggests that barriers to accessing PrEP and gaps in provision still exist, at least among non-males who would benefit from PrEP.

## 1 Introduction

With an estimated 39.0 million people living with the human immunodeficiency virus (HIV), 1.3 million new infections and 630,000 deaths in 2022, HIV remains a severe global public health challenge (1). Germany is considered a low prevalence country, with an estimated 96,700 people (0.1% of the country’s population) living with HIV in Germany by the end of 2023 (2). However, transmission of the virus remains an issue with approximately 2,000 cases per year in recent years. While 72% of people living with HIV are men who have sex with men (MSM), the number of new infections among MSM has declined since 2007. In contrast, the number of new HIV infections among heterosexuals and people who inject drugs is stable or increasing and they are now accounting for almost 50% of new HIV infections in Germany (2).

The favorable development in the group of MSM can be attributed to improvement in testing opportunities and willingness, as well as early initiation of antiretroviral therapies (ART) that prevent the development of acquired immunodeficiency syndrome (AIDS) and transmission of the virus (3). In recent years, HIV pre-exposure prophylaxis (PrEP) has been added as a new prevention tool. In 2016, tenofovir disoproxil fumarate and emtricitabine (TDF/FTC), previously established as a component of antiretroviral therapy (ART) was approved by the European Medicines Agency as the first daily oral PrEP. Since then, it is legally available as an effective agent for the prevention of HIV in Germany to HIV-negative adults who are at high risk of infection (4).

While PrEP was initially associated with high costs, the introduction of generics in 2017 and the integration into the coverage of the German statutory health insurance (SHI) in September 2019 reduced barriers to access (3, 5, 6). SHI in Germany provides full coverage of all medically necessary healthcare. This includes general practitioners, specialized and inpatient care as well as prescription of medication, like PrEP. Prescriptions of any kind usually call for a small supplementary charge of 5–10 EUR at pick up in the pharmacy (7, 8). Besides medication, SHI PrEP coverage incorporates counseling, initiation and monitoring including testing for HIV and other sexually transmitted infections (STI) by specialized providers (6). PrEP is approved for daily use, but there is growing evidence of divergent non-daily intake schedules (9). The introduction of PrEP as a SHI service in Germany was scientifically monitored and evaluated by the Robert Koch Institute (RKI) as part of the EvE-PrEP project (evaluation of the introduction of HIV pre-exposure prophylaxis as a statutory health insurance benefit) (10). A monitoring and surveillance system for PrEP in Germany (surveillance of HIV-PrEP provision in Germany (PrEP-Surv)) was established at the RKI and operated until the end of 2024 (11, 12). The EvE-PrEP project conducted a claims data analysis of several health insurance providers within the initial 6 months of SHI implementation (10). However, in-depth claims data analyses beyond this timeframe are currently unavailable (10).

This study aims to provide a longitudinal analysis of HIV-PrEP care in the real-world setting in Germany between September 1, 2019 and August 31, 2023 using claims data from the SHI provider BARMER. The research questions are: What trends in uptake, discontinuation and reinitiation can be observed since the beginning of SHI coverage of PrEP? How can PrEP coverage be described based on the concept of the proportion of days covered (PDC), and what does this indicate about adherence and utilization behavior? How many HIV infections occurred within the study period? How many courses of post-exposure prophylaxis (PEP) were initiated among PrEP users before, during, between and after PrEP courses?

## 2 Methods

### 2.1 Study design and data source

This study was conducted as part of the German Federal Ministry of Health-funded and RKI lead project, “Surveillance of HIV Pre-Exposure Prophylaxis Care within the Statutory Health Insurance System in Germany” (PrEP-Surv) for which BARMER as a cooperating partner granted RKI access to the scientific data warehouse (W-DWH) (12). BARMER is the second-largest SHI provider in Germany covering 8.6 million individuals, which corresponds to approximately 12.3% of all SHI-covered people in Germany (13).

This study utilized anonymized claims data including demographics, as well as medical care data such as standardized billing codes for individual medical services (GOP), which are unique to the German healthcare system, alongside pharmacy fills, anatomical therapeutic chemical codes (ATC) and defined daily doses (DDD) per insured individual. Data were extracted using SQL queries from BARMER’s W-DWH. Descriptive statistics and further analyses on patient demographics were conducted using Microsoft Excel and R Studio (4.2.1) software.

The anonymized data from the BARMER W-DWH has been processed in accordance with the data utilization agreement between BARMER and the RKI. It complies with the social data protection requirements of the German Social Security Code (SGB), in particular SGB I §30 in conjunction with SGB X §67c and §75, as well as the European Union’s General Data Protection Regulation (GDPR). The procedure has been approved by the supervisory German Federal Social Security Office. All individuals involved in data processing have signed data protection and non-disclosure agreements. Written informed consent was waived as this study’s data were preexisting and anonymized.

This longitudinal study was based on the principles outlined in the guidelines from REporting of studies Conducted using Observational Routinely-collected Data for pharmacoepidemiology (RECORD-PE) (14) and the German Consensus on Reporting Standard for Secondary Data Analyses, Version 2 (STROSA) (15).

### 2.2 Study population and definition of PrEP, PEP and HIV infection

PrEP users were defined as individuals with at least one PrEP care-associated GOP (01920–01922, 01930–01936; see Supplementary material 1) followed by a pharmacy retrieval of at least one stand-alone ATC coding of TDF/FTC (J05AR03). PrEP initiation was defined as the date of the first pharmacy fill for a stand-alone TDF/FTC, regardless of its temporal relationship to GOP codes, as those were newly established at the start of SHI PrEP coverage and thus not yet consistently used.

Our study population includes all BARMER-insured individuals who initiated PrEP between September 1, 2019 and August 31, 2023. We extracted demographic information including dates of birth and death, sex, place of residence at first PrEP initiation. To gain insight into PrEP care we extracted prescription redemption date and prescribed DDD, (re-)fills and any additional ART drugs to investigate PEP courses as well as HIV infections during the study period.

When individuals received any ART other than TDF/FTC for ≤ 35 days this was categorized as PEP. A course of PEP could take place at any time within the observation period before, during, between or after a course of PrEP. Additionally, we extracted HIV infections during the study period and assessed them using only ATC codes as we found International Classification of Disease (ICD) codes unreliable due to frequent miscoding in the dataset. Claims data analysis in EvE-PrEP showed that ICD-codes have been falsely used to account for HIV testing during PrEP instead of secured HIV diagnoses and the HIV guideline calls for immediate initiation of ART when an infection is diagnosed (10, 16). ATC coding of any ART other than TDF/FTC prescribed for > 35 days was classified as a treatment for HIV infection. The day of HIV treatment initiation was defined as HIV manifestation. Individuals classified with treatment for HIV infection previous to receiving stand-alone TDF/FTC were excluded.

### 2.3 Discontinuation

Discontinuation was defined as the day of the last pill remaining after the last pharmacy refill when no refill occurred within 120 days (17, 18). A stand-alone TDF/FTC refill, after surpassing the permissible gap of 120 days, was considered a reinitiation and marked the start of a new PrEP course. PrEP initiations, discontinuations and reinitiations, as described, were analyzed by calendar quarter. Due to the end of observation on August 31, 2023, we included pharmacy fills until December 31, 2023. This allowed for 120 days of follow-up to draw definitive conclusions about whether or not PrEP was discontinued by the end of observation. Individuals who had remaining pills as of December 31, 2023 were considered to be on PrEP by the end of the observation period. Other events were not considered for this follow-up period.

A Kaplan-Meyer survival analysis was conducted to visualize the time until discontinuation of the first PrEP course.

When HIV infection or death occurred before the calculated date of discontinuation, the respective date was considered as discontinuation. Given the young age of PrEP users (see Table 1) it is likely that users were sexually active until the end of their lives and continued to use the drug until then.

**Table 1:** Characteristics of BARMER insured PrEP users between September 2019 and December 2022.

Characteristics	
Total study population, N (%)	5,208 (100%)
Age (years) at time of first PrEP initiation, N (%)	
16–19	70 (1.3%)
20–29	1,526 (29.3%)
30–39	1,844 (35.4%)
40–49	1,015 (19.5%)
50–59	596 (11.4%)
≥ 60	157 (3.0%)
Sex, N (%)	
Male	5,137 (98.6%)
Female	70 (1.3%)
Divers	1 (<0,1%)
No. of PrEP courses initiated, N (%)	
1	5,208 (100%)
2	1,392 (26.7%)
3	408 (7.8%)
4	99 (1.9%)
5	8 (0.2%)
6	1 (<0.1%)
Total	7,116
HIV infections, N (%)	6 (0.1%)
Deaths, N (%)	17 (0.3%)
No. of PEP courses initiated, N (%)	267* (100%)
Before first PrEP initiation	197 (73.8%)
During PrEP course	23 (8.6%)
Between PrEP courses	28 (10.5%)
After last PrEP stop	19 (7.1%)
Number of PrEP users per 100.000 inhabitants by population size	
Population size <100,000	1,406 (2.4 per 100,000)
Population size 100,000 - < 250,000	442 (5.9 per 100,000)
Population size ≥250,000 - < 500,000	343 (9.1 per 100,000)
Population size ≥500,000 - < 1,000,000	528 (8.8 per 100,000)
Population size ≥1,000,000	2,412 (25.6 per 100,000)
Population size Unknown	77

*Initiated by 231 individuals.

### 2.4 Assessment of PrEP coverage

**Figure 1:**
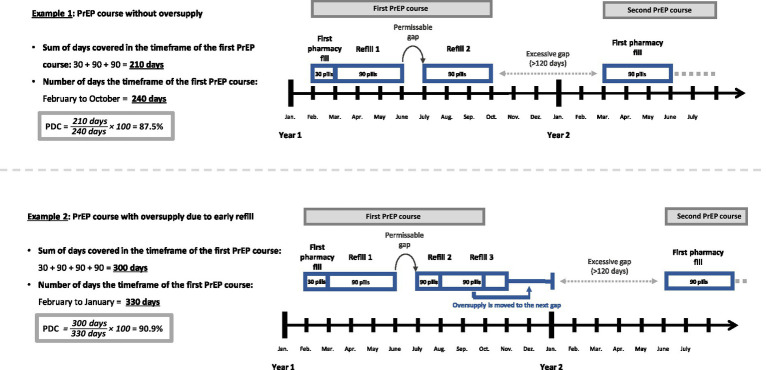
Illustration of the method of the Proportion of Days Covered (PDC)*. *for better understanding this simplified illustration assumes that each month consists of 30 days.

As recommended by the Pharmacy Quality Alliance we chose the Proportion of Days Covered (PDC) as a metric of coverage to estimate PrEP adherence (19). While PDC cannot confirm whether a medication was taken as prescribed, it offers insight into the drug’s availability to an individual based on pharmacy fills (20). To calculate the PDC we defined timeframes of PrEP courses at the individual’s level (see Figure 1) (20, 21). The timeframe between initiation and discontinuation, as defined above, marks one course of PrEP. Subsequently, a refill after surpassing the permissible gap of 120 days was considered as reinitiation and would mark the start of a new PrEP course. Therefore, the excessive gap-time (>120 days) between PrEP courses is not part of the PDC calculation for PrEP courses.

Additionally, we calculated a total PDC for individuals between their first initiation to their last discontinuation, which in contrast to the PDC of PrEP courses, did include excessive gaps. The PDC was calculated for courses/individuals with at least two pharmacy fills because PrEP courses with only one fill per definition have 100% coverage and were therefore excluded.

PDC is calculated as follows (20):


$$\mathrm{PDC}=\frac{\mathrm{Sum}\;\text{of days covered in timeframe}\;}{\text{number of days in timeframe}}\times 100$$

What sets the PDC apart from other frequently used methods like the Medication Possession Ratio (MPR) is its consideration of the date of the (re-)fill (20, 21). For a day to be covered the prescription must have been filled on this day or in the past. It also assumes that medication use follows the prescribed DDD, which for PrEP is one pill per day (22). Any oversupply of pills due to early refills is shifted to the next gap (see Example 2; Figure 1). Consequently, the PDC cannot exceed 100% of coverage. MPR, in contrast, does not factor in the date of the refill. It sums up all pill supply within the timeframe and divides it by the number of days in the timeframe. This may lead to an overestimation of adherence due to early refills (20, 21). Example 2 reveals the difference between the two methods. As oversupply is simply summed up rather than shifted into the future, the timeframe for MPR remains constant, terminating with the last refill by November (270 days). While PDC and MPR would produce the same result in example 1, example 2 would lead to a MPR of 111.1% (300 DDD / 270 days) despite the gap of 1 month.

We tested the association between individuals’ total PDC (below average, as determined from the data) and the factors age, place of residence at first PrEP initiation (classified by population size), and length of PrEP use. This was done using a univariable chi-square test and a multivariable logistic regression model, adjusted for these variables.

## 3 Results

### 3.1 Population

Based on the study’s inclusion and exclusion criteria we identified 5,208 individuals who initiated PrEP between September 1, 2019 and August 31, 2023. The total observation time was 8,822 person-years (py) with a median and average PrEP observation time of 1.3 py (IQR 0.5–2.9 py; range 0–4 py) and 1.7 py (SD 1.3 py, range 0–4 py), respectively. A total of 17 (0.3%) deaths occurred.

Characteristics of the study population are summarized in Table 1. The median age of PrEP users at the time of first initiation was 34 years (IQR 28–43) and almost all of them were male (98.6%). The youngest PrEP user was 17 and the oldest was 80 years old. Almost half of all PrEP users (*N* = 2,412, 46.3%) were based in an area with ≥1 million inhabitants at the time of their first PrEP initiation, while 27% (*N* = 1,406) in an area with <100,000 inhabitants. When classified by the number of PrEP users per 100,000 inhabitants most PrEP users were found in regions with ≥1 million inhabitants (25.6), followed by areas with ≥250,000- < 500,000 inhabitants (9.1) and ≥500,000- < 1,000,000 inhabitants (8.8).

The study population of 5,208 individuals initiated a total of 7,116 PrEP courses within the observation period. Of the study population 26.7% (*N* = 1,392) initiated a second course, a further 7.8% (*N* = 408) started a third course, 1.9% (*N* = 99) a fourth course, 0.2% (*N* = 8) a fifth course and 0.02% (*N* = 1) a sixth course.

### 3.2 PrEP initiations and discontinuation

**Figure 2:**
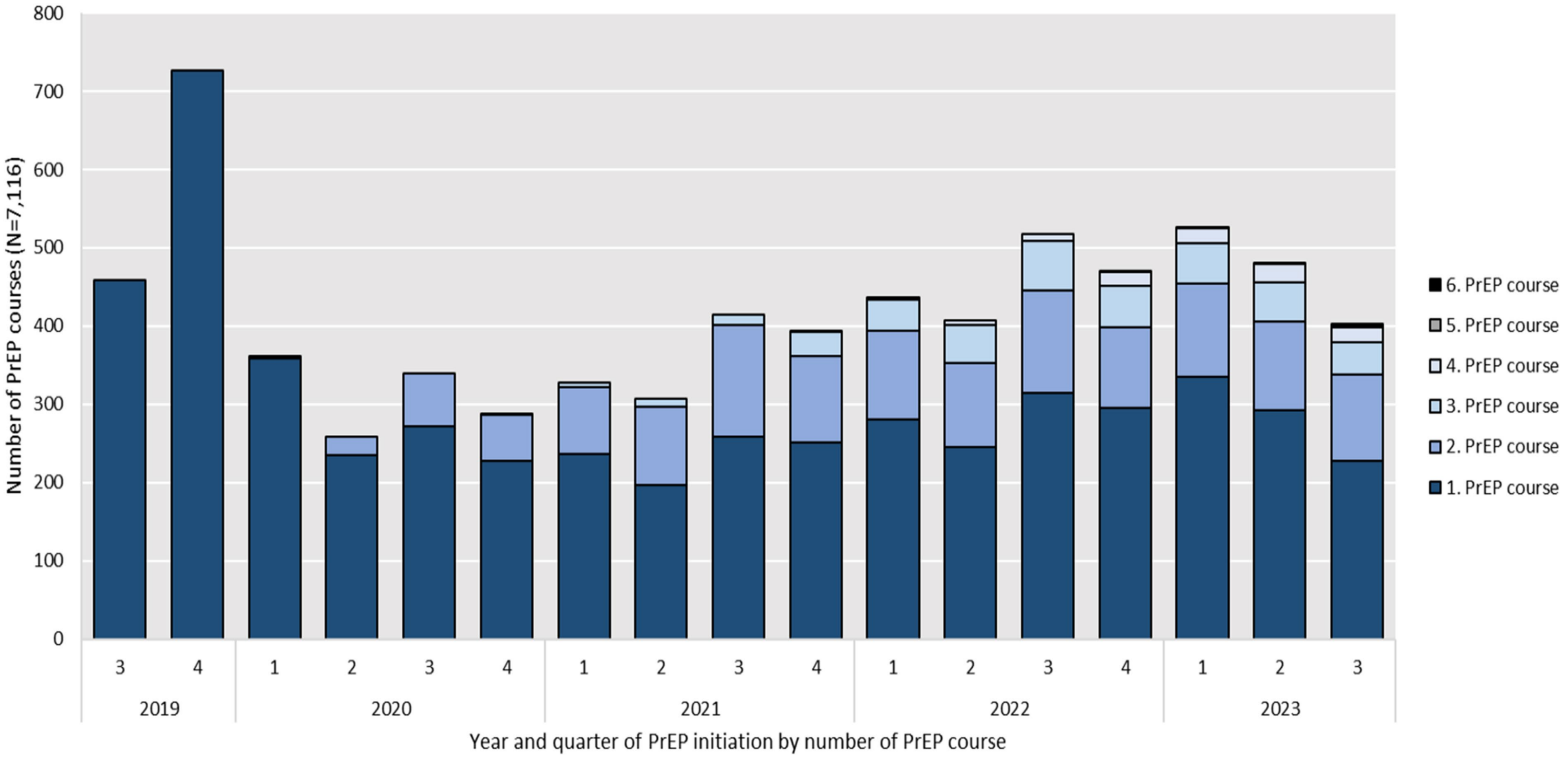
Year and quarter of PrEP initiation, including reinitiations by number of PrEP course between September 2019 and August 2023 (*N* = 7,116).

Figure 2 illustrates the number of PrEP initiations by number of PrEP courses per quarter and year over the study period. It shows that every year the vast majority of initiations stem from individuals starting their first PrEP course under BARMER coverage in the observation period. Out of the total 5,208 PrEP users observed, 22.7% (*N* = 1,184) began their first PrEP initiation shortly after SHI coverage was introduced in 2019. In the following years the number of first PrEP initiations decreased to 1,093 in 2020 and 942 in 2021. In 2022 first PrEP initiations increased again to 1,134. As of August 31, 2023, the number of first PrEP initiations was 855. Assuming the same dynamic, the number of first PrEP initiations would rise to an estimated 1,140 by the end of 2023.

Reinitiations of consecutive courses (numbers 2 to 5) after previous discontinuation made up 12.4% (*N* = 155) of total initiations in 2020 and rose to 34.7% (*N* = 500) in 2021, to 38.1% (*N* = 697) in 2022 and 39.4% (*N* = 556) in 2023.

The annual number of total initiations, including PrEP reinitiations increased from 1,248 courses in 2020 to 1,442 in 2021 and 1,831 in 2022. As of August 31, 2023, the number of total PrEP initiations, including PrEP reinitiations was 1,411. Assuming the same dynamic, this number would rise to an estimated 1,881 by the end of 2023. The lowest numbers of both first PrEP initiations and total PrEP initiations including PrEP reinitiations per quarter were seen in the second and fourth quarter of 2020 which overlap with the first and second national COVID-19 lock-down in Germany.

**Figure 3:**
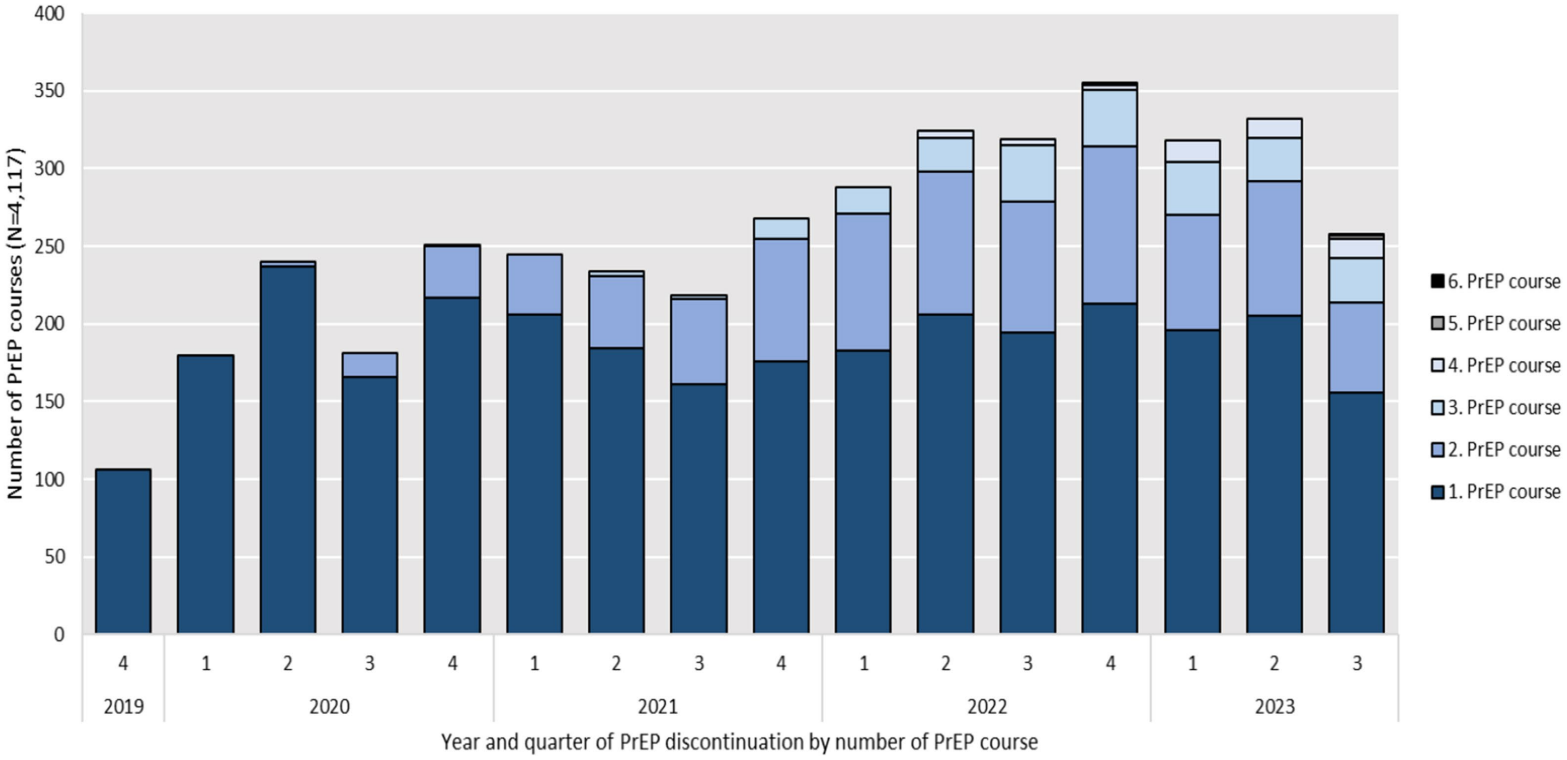
Year and quarter of PrEP discontinuation by number of PrEP course between September 2019 and August 2023 (*N* = 4,117).

Figure 3 shows the number of PrEP discontinuations per calendar quarter. Discontinuations (*N* = 4,117), based on the defined criteria of more than 120 days without PrEP on hand, occurred throughout the entire study period. As the number of PrEP initiations increased over time, so did the number of discontinuations from 106 in 2019, to 852 in 2020, 965 in 2021 and 1,286 in 2022. As of August 2023, the number of discontinuations was 908. Assuming the same dynamic, the number of PrEP discontinuations would rise to an estimated 1,211 by the end of 2023. Throughout the study period, a notable decrease in PrEP discontinuations was observed during the third quarters of 2020 and 2021, coinciding with the lifting of COVID-19 restrictions.

**Figure 4:**
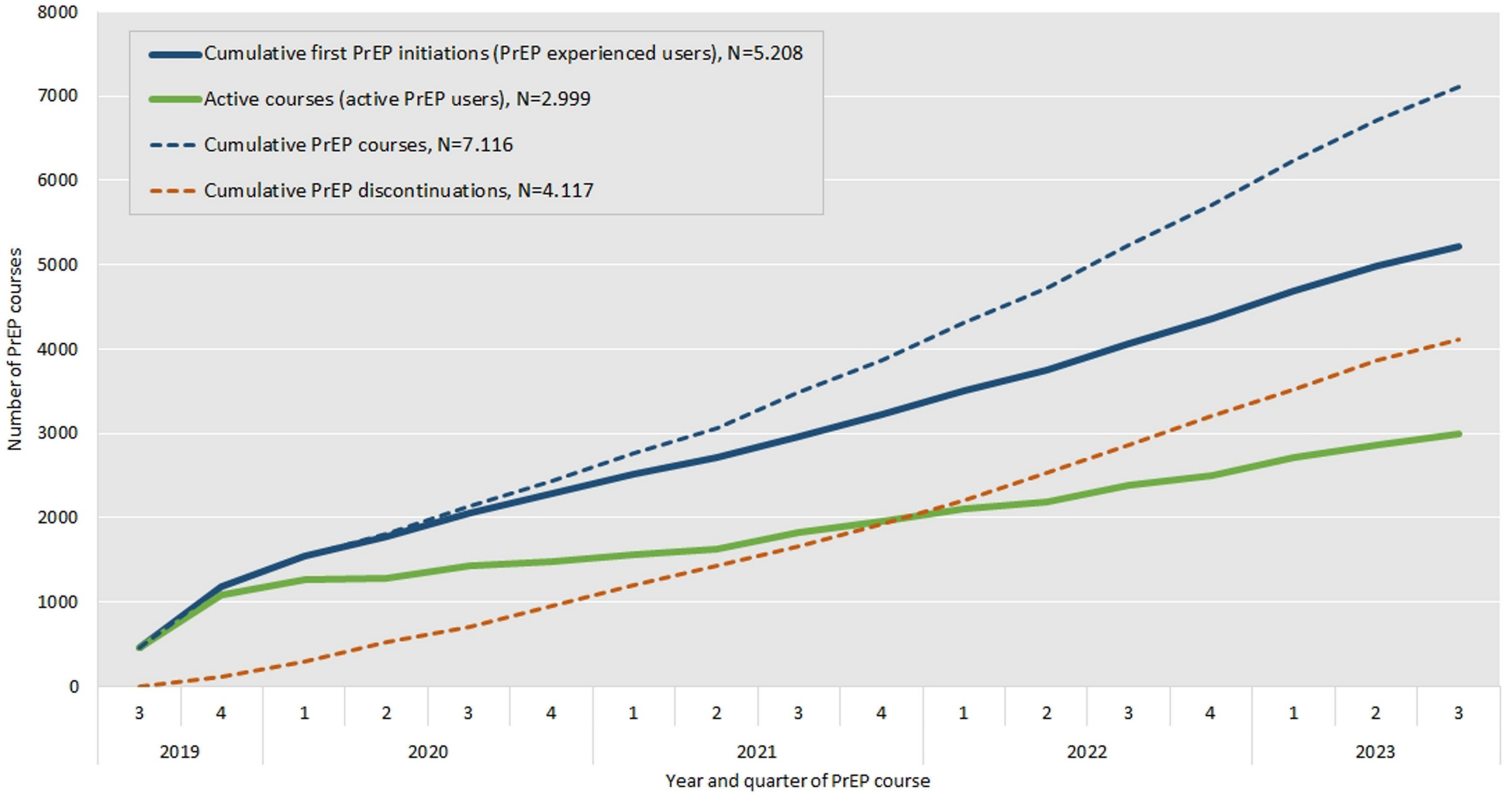
Number of cumulative first PrEP initiations or PrEP experienced users (bold blue line), cumulative PrEP courses ever started (doted blue line), cumulative PrEP discontinuations (doted red line) and resulting number of active PrEP users (bold green line) by year and quarter between September 2019 and August 2023.

Figure 4 illustrates the relation between the number of first PrEP initiations which corresponds to the number of experienced PrEP users (bold blue line) as well as the number of active PrEP users (bold green line) that results from the difference of the number of cumulative PrEP courses ever started (doted blue line) and the cumulative number of PrEP discontinuations (doted red line) at each quarter. The number of first PrEP initiations shows a steady increase over time, with a more pronounced rise in later years. The number of active PrEP users demonstrates initial growth, followed by slower increases, particularly during the second and fourth quarters of 2020 and the first and second quarters of 2021, likely due to the impact of the COVID-19 pandemic. After mid-2021, the number of active PrEP users increases again. Both cumulative PrEP courses and cumulative PrEP discontinuations show a consistent rise.

Considering all PrEP courses observed, a total of 2,999 (58%) PrEP users were on PrEP by the end of the study and were therefore considered to be active PrEP users. A total of 2,222 (43% of the study population) were still on their first course.

**Figure 5:**
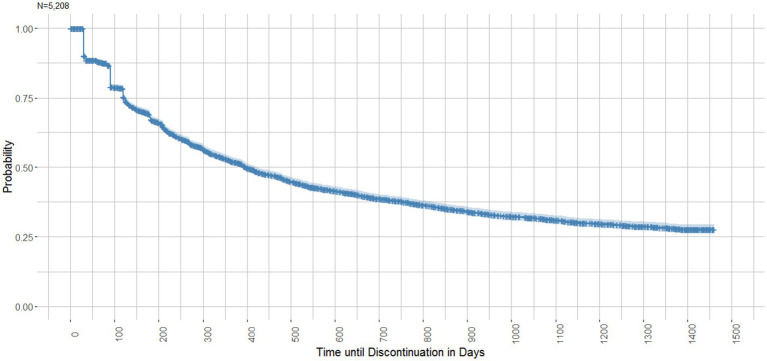
Kaplan–Meier survival curve: Time until discontinuation of the first PrEP course.

Figure 5 illustrates the time until discontinuation of the first PrEP course (*N* = 2,986) in a Kaplan–Meier survival curve. The median Kaplan–Meier survival time until discontinuation was 397 days (IQR 383–421 days). Three drops in the survival curve are noticeable. After (30–35 days) 11.2% of courses were discontinued, another 7.9% ended after ~90 days and a further 4.3% ended after ~125 days, reflecting PrEP package sizes. The survival probability by the end of the observation period was 27.7% (95% CI 26.2–29.4).

### 3.3 PrEP coverage

Total PDC was calculated for individuals (between their first initiation to their last discontinuation) as well as PrEP courses when at least two pharmacy fills occurred. Individuals or courses with only a single fill were excluded because, as by definition, this would result in a 100% coverage. Therefore, a total of 743 individuals were excluded from the analysis for individuals total PDC and 2,105 PrEP courses were excluded from the analysis of PrEP coverage for the PDC of all courses.

The median PDC of all observed courses was 91.3% (IQR 78.9–98.6%). While the median coverage of the first PrEP course was highest, it decreased for the second and subsequent courses (see Table 2).

**Table 2:** Proportion of days covered (PDC) for courses/individuals with at least two pharmacy fills.

	Total PDC of individuals (*N* = 4,466)	PDC of all courses (*N* = 5,011)	PDC of first courses (*N* = 4,031)	PDC of second courses (*N* = 724)	PDC of third courses (*N* = 209)	PDC of fourth courses (*N* = 43)	PDC of fifth courses (*N* = 4)	PDC of courses with add-on PEP (*N* = 21)
Median	85.4%	91.3%	92.8%	84.3%	76.7%	76.3%	66.4%	79.1%
IQR	61.1–97.4%	78.9–98.6%	82.0–99.0%	71.2–94.7%	66.2–90.5%	65.8–98.3%	43.7–89.1%	71.9–88.1%
Min	2.9%	25.0%	25.0%	26.2%	30.2%	34.3%	33.3%	39.2%
Max	100%	100%	100%	100%	100%	100%	100%	100%
Mean (SD)	76.5% (24.4)	86.9% (13.9)	88.5% (12.8)	81.5% (15.6)	76.7% (16.3)	77.7% (19.8)	66.5% (27.2)	77.8% (13.8)

**Figure 6:**
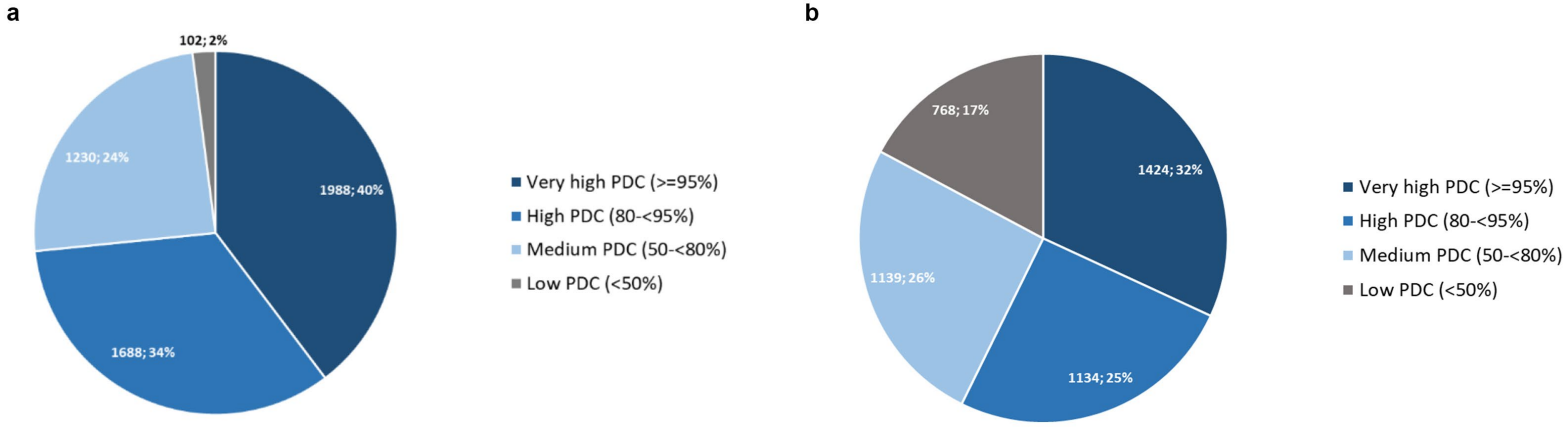
**(a)** PDC of all PrEP courses with at least two pharmacy fills (*N* = 5,011); **(b)** PDC of individuals with at least two pharmacy fills from first initiation until last discontinuation (*N* = 4,465).

Figure 6a shows that the majority (74%) of all courses showed very high or high PrEP coverages. Very high PDC, suggesting daily use were seen in 40% of all PrEP courses and only a minority (2%) of courses had low coverage of less than 50%.

We found a total median PDC for individuals (*N* = 4,465) between their first initiation to their last discontinuation of 85.4% (IQR 61.1–97.4%). The proportion of individuals with very high or high PrEP coverages was 57% (see Figure 6b).

The univariable and multivariable test of association between individuals total PDC below average (as determined in the data with a median total PDC of 85.4%) and age, place of residence (classified by population size) and length of PrEP use showed an association between individuals total PDC and place of residence as well as with the length of PrEP use in the chi square test. However, the logistic regression model only confirmed the association of decreasing individuals total PDC with increasing length of PrEP use (see Supplementary material 2).

### 3.4 Post-exposure prophylaxis

As shown in Table 1, we recorded a total of 267 prescription retrievals for PEP among 231 individuals between September 1, 2019 and August 31, 2023. Prior to PrEP initiation, 197 courses of PEP (74% of total PEP events) were retrieved, 19 courses of PEP (7%) took place after individuals had discontinued PrEP. Further, 23 courses of PEP (9%) were administered during active PrEP courses based on our definition of initiation and discontinuation and 28 courses of PEP (10%) took place between PrEP courses. The median PDC of active PrEP courses with add-on PEP was 79.1% (IQR 71.9–88.1%) ranging from a minimum of 39.2% to a maximum pill coverage of 100%. Low pill coverages of less than 50% were rare at 4.5%.

### 3.5 HIV

A total of six HIV infections were detected among the study population within the observation period September 1, 2019 to August 31, 2023. HIV infections occurred in 2020 (*N* = 1), 2022 (*N* = 2) and 2023 (*N* = 3). The HIV incidence rate was 0.068/100 py. All individuals affected were male and none of them received a course of PEP within the timeframe of the study. One case was within the 20–29 years of age range, three were within the 30–39 years of age range and two were within the 50–59 years of age range when initiating PrEP. Two people lived in areas with ≥1 million inhabitants, one in an area of 250,000– < 500,000 inhabitants, one in an area of 100,000- < 250,000 inhabitants, one in an area of less than 100,000 inhabitants and one in an area of unknown population size when initiating PrEP.

None of them had more than one course of PrEP. PrEP courses consisted of 1, 2, 2, 3, 8 and 11 pharmacy fills, respectively, covering 5, 15.6, 28.9, 35.9, 123.9 and 192.4 weeks on PrEP.

Five out of the six HIV cases occurred after discontinuing PrEP. They were diagnosed in the context of counseling and laboratory testing associated with PrEP reinitiation. The gaps between discontinuation and HIV manifestation were 16.7, 47.7, 66, 68.1 and 118.6 weeks. PDC of prior courses with more than one pharmacy fill were medium to high (64.2, 77.4, 83.7, 89.1%). Four of them had PrEP care-associated appointments after discontinuation, including counseling and laboratory testing before their HIV manifestation but no refills took place.

One HIV infection was detected during an active PrEP course based on our definition which had a PDC of 74.2%. This person retrieved the most pharmacy fills of all HIV cases in this study and spent a total of 3.7 years on PrEP. He was found to be HIV positive few days after the last PrEP appointment including counseling, laboratory testing and a PrEP refill. Before this last appointment and refill this person had a calculated gap of 111 days which is just about permissible according to our definition.

All cases immediately initiated ART following counseling and laboratory testing for HIV.

## 4 Discussion

### 4.1 Population

To the best of our knowledge, this is the longest claims data analysis on PrEP use in Germany conducted to date. Based on PrEP associated GOP and ATC codes, we detected a total of 5,208 BARMER insured PrEP users between the introduction of PrEP as a SHI service in September 2019 and the end of the study period in 2023. Characteristics of the study population show similarities to those of previous studies in Germany (23) as well as claims data studies from other countries and health care systems like the US, France and Australia (17, 18, 24–26).

The median age of our study population at the time of first initiation was 34 (IQR 28–43) which is comparable with previous research in Germany (38 years) (9) and studies on PrEP users in France (36 years) (24) and Australia (33 years) (26). In some studies, in the US, PrEP users seem to be slightly older (mean age between 33 years and 41 years) (17, 18, 27) which may be due to a prolonged availability of the drug there.

PrEP users in our dataset were predominantly male (98.6%). From previous studies using data from German HIV-specialty centers, we know that the majority (up to 99%) of PrEP users are MSM (9, 23, 28). The proportion of MSM in this dataset is expected to be similar but possibly slightly lower as the claims data is expected to reflect other certified providers as well. Other institutions assumingly have different patient characteristics, as HIV-specialty centers are less common in rural areas and attract a specific group of patients, mostly MSM (9, 10). Further analyses could potentially provide a more detailed look into differences according to provider characteristics.

The proportion of 1.4% females in our dataset was comparable to previous studies in Germany (9, 28). Studies in countries like France, United States and Australia where PrEP availability or coverage has been established for a longer period of time show higher proportions of around 2.5–4.9% of female PrEP users (17, 24–27). A continuous monitoring would be beneficial to assess whether the proportion of women using PrEP in Germany increases over time.

Almost half of all PrEP users lived in areas with a population size of more than 1 million at the time of their first PrEP initiation, and these areas also had the highest number of PrEP user per 100,000 inhabitants. While more than a quarter of PrEP users lived in areas with a population of less than 100,000, the number of PrEP users per 100,000 inhabitants in these areas was only a tenth of that in areas with a population of more than 1 million (2.4 vs. 25.6). This is comparable to findings from claims data analyses in EvE-PrEP (10) as well as to findings from a study on the dynamics of HIV-PrEP use and coverage in Germany that suggested low PrEP coverages in some federal states (29). The distribution of PrEP users across Germany is likely to be largely influenced by the availability of providers, including HIV care centers and community counseling centers. Overall, our study confirms previous results and the finding of low PrEP coverages and assumingly unmet needs in particularly rural areas (29).

Overall, it is noteworthy that our sample of BARMER-insured individuals might not be representative of all PrEP users in Germany as the composition of insurance holders varies between different health insurance providers (10). Furthermore, this longitudinal study only provides insight into BARMER-insured individuals who, in addition to the drug, also received PrEP counseling, care or testing at least at some point in time according to the PrEP guideline. Due to the inclusion criteria, non-guideline-based PrEP courses that were never accompanied by a PrEP counseling, care or testing billing code were not captured. We assume the number of PrEP users to whom this applies over the entire observation period is small and data from HIV specialists support our findings (11).

Previous studies have shown barriers to access to PrEP care in Germany, especially for persons outside of MSM communities, including women, migrants, sex workers, injecting drug users, trans, non-binary or gender diverse persons, people without health insurance and people in rural areas, many of whom could potentially benefit from PrEP (10). Structural barriers often play a particularly significant role for those groups. As studies have shown the intersection of health insurance design and preventive care utilization, underscores that structural determinants are closely aligned with challenges in healthcare access, adherence, and population targeting (30–32). The data from our study support previous findings of high PrEP demand in men, assumingly mostly MSM and suggest that there are still barriers to accessing PrEP and gaps in provision, at least among non-males with PrEP need.

### 4.2 PrEP initiations and discontinuation

The number of first observable PrEP initiations were highest in 2019, slightly decreased in 2020 and 2021 but increased again 2022 and, assuming the same dynamic of PrEP initiations, would increase further in 2023. The number of total annual initiations increased over the observation period but was driven by reinitiations (see Figure 4). This might be related to PrEP discontinuations and fewer initiations during the COVID-19 pandemic. Almost 25% of the study population started a second PrEP course after discontinuation according to our definition. Third, fourth or fifth PrEP courses were rare at around 7%. Surveys in HIV-specialty centers have reported lower PrEP demand in times of contact restrictions during the COVID-19 pandemic (33) and pharmacy prescription data also showed effects on PrEP uptake (29, 34). The rollout of SHI-covered PrEP in Germany largely coincided with the beginning of the COVID-19 pandemic, which significantly influenced healthcare access and patterns of health-seeking behavior. In our quarterly assessment of initiations and discontinuations, this may be reflected in lower PrEP uptake in Q2 of 2020 as well as Q4 of 2020 and Q1 and Q2 of 2021 (see Figure 2) and in lower number of discontinuations in Q3 of 2020 and 2021, when COVID-19 restrictions were partially lifted (see Figure 3). Overall, no declines in PrEP uptake were observed. On the contrary after fewer first initiations in 2020 and 2021, which could be related to the COVID-19 pandemic, we observed an increase again in 2022. This supports findings from other German studies that PrEP needs are not yet covered in Germany (29, 35).

Interestingly, a large proportion, 23% (*N* = 1,184), of all observed PrEP courses began within the first 4 months after the SHI coverage was introduced in 2019. This indicates a high level of awareness within the community of people at high risk for HIV infection, assumingly mostly MSM, to the change in health care policy and PrEP as a new SHI service.

The data provided by SHI does not allow for conclusions to be drawn regarding the number of individuals who previously used PrEP on a self-payer basis, who directly benefited from SHI coverage due to the reduction of the financial burden. A relevant portion of initiations, particularly at the start of the observation period and beginning of SHI PrEP, are assumed to be continuations of previously self-paid PrEP. Previous findings from HIV-specialty centers suggest that up to 53% of PrEP users in 2020 started the drug before September 2019 (28).

Considering discontinuations, the second (+33.5%) and third quarter of 2020 (+39.7%) showed increasing numbers of discontinuations compared to the respective previous quarter which reflect times of lockdown and contact restrictions in Germany. However, the increase in discontinuations over time, based on the defined criteria of more than 120 days without PrEP, should not in itself be interpreted as alarming. This is to be expected with rising initiations. Further, PrEP is not designed for permanent use when the need for HIV prevention no longer persists or other preventive measures are taken. Additionally, a narrower definition of the permissible gap probably would have resulted in higher variances. Further, it is inherent that the likelihood of observing PrEP reinitiations decreases toward the end of the observation period. The median and average duration of PrEP use were 1.3 and 1.7 person-years, respectively. Therefore, it is expected that discontinuations will accumulate over a longer observation period. Eventually, a steady state may be reached, with individuals at risk of HIV starting PrEP, while those without risk discontinuing it. This steady state would ultimately result in a high cumulative protective effect.

The trajectory of PrEP initiations, discontinuations and the resulting active PrEP users showed a steady overall increase (see Figure 4). The steeper rise in first PrEP initiations in later years suggests ongoing growth in PrEP demand and uptake. This trend aligns with analyses of pharmacy prescription data, which demonstrate a continuous increase in the number of PrEP users between September 2019 and the end of 2023 (36). The lower numbers of active PrEP users observed during 2020 and 2021 are likely related to the COVID-19 pandemic, as discussed earlier. The consistent rise in cumulative PrEP courses and cumulative PrEP discontinuations reflects the accumulation of new PrEP courses initiated over time. However, it also highlights that while new users continue to initiate PrEP, a substantial proportion discontinues it. These patterns underscore the importance of monitoring both PrEP uptake and retention. Addressing the reasons for discontinuations is critical to optimizing the overall effectiveness of PrEP as a public health intervention for HIV prevention.

The Kaplan-Maier survival analysis revealed a median time until discontinuation of the first PrEP course of 397 days (IQR 383–421 days). The survival curve shows two small drops relatively early after initiation (see Figure 5). After 35 days 11% of courses were discontinued and after 12.9 weeks (~90 days) another 8% ended. These survival times correspond with PrEP package sizes which are usually 30/35 for PrEP initiation and 90 pills for continued PrEP. Therefore, these early discontinuations can be attributed to individuals with one pharmacy fill only and no refills within the permissible gap. However, individuals who stay on their first PrEP course, specifically those who are active users after 52 weeks (survival probability of 52%) appear to be more persistent as the survival curve flattens over time.

A remarkable 43% of individuals were still on their first PrEP course by the end of the study which indicates a high level of commitment for some long-term users to PrEP. When considering all PrEP courses, more than half were active PrEP users (*N* = 2,999, 58%) by the end of the study. Assessments from pharmacy prescription data revealed a total of around 40,000 PrEP users in Germany at the end of 2023 (29, 36). Assuming the rate of active PrEP users to those with PrEP experience also applies to the total PrEP use shown in pharmacy prescription data, this would approximate to around 70,000 people with PrEP experience in Germany at the end of 2023. Nevertheless, caution is required when generalizing these results to all PrEP users in Germany.

### 4.3 PrEP coverage

Overall PrEP coverage was high, at a median PDC of 91.3% of all observed courses and 85.4% of all observed individuals from first initiation until last discontinuation (individuals total PDC). Very high coverages, resembling a daily intake regimen as proposed by the German and Austrian PrEP guideline, were found in around one-third of courses and individuals. Around two-thirds of courses and individuals still had high pill coverages, which are known to provide high PrEP efficacy in MSM, which presumably make up most of our study population (29, 35, 37) and potentially even in women (38). Furthermore, the negative association between the number of PrEP courses and PDC, as presented in Table 2, stands out. While the median coverage in the first course was high it dropped to medium for subsequent courses which may indicate decreasing adherence after each previous discontinuation. However, the number of PrEP users with more than two courses was low. In general, it should be noted that the PDC is only an approximation of adherence. It gives insight into a person’s PrEP availability, which is why we chose the collection date rather than the prescribing date. However, it remains uncertain whether or how the collected pills are taken. Pill sharing with other people, possibly without health insurance, may occur as well as pill loss or discard.

Medium or low coverages may represent low adherence in daily PrEP or possibly event-driven PrEP use. Event-driven PrEP may be prescribed as such by healthcare providers off-label or could be independently applied by users themselves. Accurate knowledge about divergent non-daily PrEP intake schemes is required to achieve effective HIV prevention but may provide sufficient protection when applied correctly (39–41).

The lower median PDC of consecutive courses could also reflect a low coverage over the total duration of an individual’s PrEP experience, which due to a violation of the permissible gap, would, possibly falsely, be displayed as a series of several courses. We chose a relatively long permissible gap of ≤ 120 days to avoid overestimation of discontinuations and reinitiations. Still, the course based-view itself facilitated the detection of discontinuations and reinitiations and our results may only approximate their true value. Therefore, in addition to calculating the PDC of courses, the total PDC for PrEP users with at least two pharmacy fills, from their first initiation to their final discontinuation, was calculated and found to be marginally lower. Although gaps between PrEP courses are included in the total PDC as PrEP time, which could potentially reduce the PDC, this only slightly affected the total PDC. Overall, both approaches resulted in comparably high coverages (see Table 2; Figure 6). As observed in the analysis of PDC for PrEP courses, the adjusted multivariable logistic regression confirmed an association between individuals’ total PDC and the length of PrEP use, demonstrating that as the length of PrEP use increased, total PDC decreased. Although the univariable analysis found an association between total PDC and place of residence, this association, along with all other factors, was not confirmed in the multivariable analysis.

The total mean PDC based on claims data in our study was 77% (total PDC) and 87% (course based PDC) and is comparable to findings from the EvE-PrEP project, which found a mean MPR of 85% with event-driven PrEP intake in ~20% of users at HIV-specialty care centers (28) and a mean MPR of 87% in the claims data analysis (10). Studies conducted in the United States showed similar results. For instance, a mean PDC of 83% was found in veterans (27) and a mean MPR of 92% was detected using claims data from a health insurance provider (17). Although PDC and MPR are not directly comparable comparison of those studies to our findings shows similar results.

### 4.4 Post-exposure prophylaxis

Most PEP retrievals (74%) took place before PrEP was initiated which suggests that the HIV risk was recognized in these individuals as PrEP was prescribed eventually. While some people had more than one PEP course, still 3.5% (*N* = 183) initiated PEP before PrEP at a time where SHI PrEP was available. It is noteworthy that the number of PEP courses dropped drastically from 197 before PrEP initiation, to only 23 as add-on PEP, 19 after PrEP and 28 between PrEP courses. This is similar to previous claims data results from Germany in EvE-PrEP (10). The median PDC of those with add-on PEP was 79.1% and therefore lower than the PDC of all PrEP courses. Still 50% of them had high coverages of at least 80%, which could provide sufficient protection (40). One individual even had a coverage of 100%. This highlights the fact that drug availability does not imply actual intake.

### 4.5 HIV

We found six HIV infections within the study population, corresponding to an incidence rate of 0.068 per 100 person-years. Overall, HIV infections in the context of PrEP use are rare which supports its high effectiveness in real-world settings (28). The longer the observation period the more HIV infections are to be expected as more people join the collective of PrEP users and possibly leave due to discontinuation (see Figure 4). This is especially the case when HIV infections occurring after discontinuation among former active PrEP users are included. We did not find patterns regarding age or place of residence.

Five people showed HIV manifestation after discontinuation of PrEP in the context of laboratory testing as part of PrEP care associated with reinitiation of the drug. PDC of their preceding PrEP courses was medium to high but below the median PDC of all PrEP courses in this study which indicates suboptimal adherence or event-driven intake schemes. Only one HIV infection was detected during an active PrEP course. This long-term users PDC of 74.2% and the large gap between the last refill and HIV manifestation indicate non-daily intake schemes or suboptimal adherence as well.

Interestingly four out of five HIV cases had PrEP care-associated appointments after discontinuation, including counseling and laboratory testing before HIV manifestation but no PrEP refills took place. While early testing could lead to false negative results, HIV was likely contracted after their last appointment and laboratory testing. It is unknown whether they were prescribed PrEP at that time but did not collect it from the pharmacy. All HIV infections were recognized in the context of PrEP monitoring and testing, which reinforces their importance. The close monitoring and support provided within the framework of PrEP led to the early detection of HIV infections. A possible limitation of our method to assess HIV through ATC codes is the fact that untreated HIV infection would not be detected. However, we assume it is highly unlikely that a person who is already in medical care and on preventive HIV medication refuses to start ART after HIV diagnosis.

## 5 Limitations

We evaluated PrEP use within the SHI system in Germany. Our observation period therefore starts with the introduction of PrEP coverage by SHI in September 2019. While PrEP has been used in Germany on a self-payer basis, and a few SHI providers had specific agreements to reimburse the drug before September 2019, BARMER insurance did not have such an agreement. The overall number of PrEP users before introduction of SHI PrEP was estimated at around 9,000 users in total (35). The regional distribution between different SHIs in Germany and in particular the number of PrEP users in the population of BARMER insured persons and the representativeness for all PrEP users is unknown. Since we used claims data of people with SHI, privately insured individuals or informal access to PrEP such as out-of-pocket or privately paid online purchase is not captured. We assume that, subsequent to the introduction of SHI-covered PrEP, only a minority of PrEP users in Germany will rely on these distribution channels. Nonetheless, we therefore avoid calculating the total number of PrEP users or the total number of HIV infections among PrEP users based on this data source. However, anonymous pharmacy prescription data, which represent the total number of persons with statutory health insurance in Germany, reflect a comparable regional distribution of PrEP utilization and trends in PrEP uptake (29). Furthermore, PrEP user characteristics from other German health insurance providers and HIV specialty care centers have been found to be similar as well (11, 42). Nevertheless, the mentioned limitations on population representativeness should be considered to avoid overgeneralization of the findings.

## 6 Conclusion

Overall PrEP coverage in this real-world study was high and PrEP proved to be highly effective with regards to the HIV incidence. Incident HIV infections almost only occurred after PrEP was discontinued. However, longer observation periods would be useful to evaluate long-term effectiveness of PrEP. PEP courses mostly took place before PrEP initiation which indicates that the HIV risk was recognized in these individuals as PrEP was prescribed eventually. Among the factors tested, we found a negative association between individuals’ total PDC and the length of PrEP use, suggesting a need for interventions to increase adherence among long-term PrEP users.

In these 4 years of observation, we saw a considerable number of PrEP discontinuations and reinitiations, as well as a duration of PrEP use that indicates that PrEP is utilized in accordance with the specific needs of the individual. On the other hand, by the end of the observation period 58% were active PrEP users and 43% were still on their first PrEP course indicating a high level of commitment to PrEP. Overall, no stagnation or decline in collective PrEP uptake was observed, suggesting that PrEP needs in Germany are not yet fully covered. This study supports previous findings of high PrEP demand in men, assumingly mostly MSM and suggests that barriers to accessing PrEP and gaps in provision identified in other studies still exist outside of MSM communities, at least among non-males who would benefit from PrEP.

Our approach of defining and analyzing PrEP use, including uptake, discontinuation and reinitiation in claims data and using PDC as an indicator of adherence as well as incident HIV infections and use of PEP can be used and adapted to other countries and health-care settings. Thereby contributing to and improving public health efforts.

## Data Availability

The data analyzed in this study is subject to the following licenses/restrictions: aggregated data supporting the conclusion of this article will be made available by the authors, upon reasonable request. Requests to access these datasets should be directed to Daniel Schmidt, schmidtd@rki.de.
