# Supplementary material for: Exploring 4 years of HIV pre-exposure prophylaxis as a statutory health insurance benefit in Germany—a longitudinal claims data analysis
Source: Front Public Health. 2025 Nov 5;13:1586736. doi: 10.3389/fpubh.2025.1586736 (PMC12626964; doi:10.3389/fpubh.2025.1586736)
Supplement: Supplementary file 1 [file Supplementary_file_1.docx]

# Supplementary Material

## Supplement 1. PrEP associated GOP codes (43)

| **GOP code** | **Description** |
| --- | --- |
| 01920 | Counseling before starting HIV pre-exposure prophylaxis (PrEP) |
| 01921 | Initiation of HIV pre-exposure prophylaxis (PrEP) |
| 01922 | Monitoring as part of HIV pre-exposure prophylaxis |
| 01930 | Creatinine in serum/plasma and eGFR calculation in the context of PrEP |
| 01931 | HIV-1 and HIV-2 antibodies and HIV-p24 antigen in the context of PrEP |
| 01932 | Detection of HBs antigen and HBc antibodies before starting pre-exposure prophylaxis |
| 01933 | Detection of HBs antibodies before the start of pre-exposure prophylaxis without documented vaccination against hepatitis B |
| 01934 | Detection of HCV antibodies in the context of PrEP |
| 01935 | Detection of treponema antibodies using TPHA/TPPA test (Lues search reaction) and/or immunoassay after individual and situational risk assessment as part of pre-exposure prophylaxis |
| 01936 | Detection of Neisseria gonorrhoeae and/or Chlamydia in pharyngeal, anorectal and/or genital swabs using the nucleic acid amplification method (NAT) after individual and situational risk assessment as part of pre-exposure prophylaxis, including pooling of materials from the swab sites if necessary |

## Supplement 2. Test of association between individuals' total PDC (below average, as determined from the data) and the factors age, place of residence at first PrEP initiation (classified by population size), and length of PrEP use.

**Adjusted multivariable logistic regression**

| **Log regression** | **Odds ratio** | **95% confidence interval** | | **P value** |
| --- | --- | --- | --- | --- |
| (Intercept) | 0,025 | 0,009 | 0,071 | 0,000 |
| **Age (years) at time of first PrEP initiation** |  |  |  |  |
| 16 - 19 | Ref. |  |  |  |
| 20 - 29 | 1,281 | 0,549 | 2,978 | 0,563 |
| 30 - 39 | 0,921 | 0,396 | 2,134 | 0,848 |
| 40 - 49 | 0,785 | 0,335 | 1,834 | 0,574 |
| 50 - 59 | 1,045 | 0,440 | 2,473 | 0,919 |
| ≥60 | 0,939 | 0,367 | 2,397 | 0,895 |
| **Place of residence at first PrEP initiation**  **(classified by population size)** |  |  |  |  |
| Population size <100,000 | Ref. |  |  |  |
| Population size 100,000 - <250,000 | 1,125 | 0,640 | 1,993 | 0,684 |
| Population size ≥250,000 - <500,000 | 1,069 | 0,605 | 1,904 | 0,818 |
| Population size ≥500,000 - <1,000,000 | 1,326 | 0,765 | 2,316 | 0,316 |
| Population size ≥1,000,000 | 1,483 | 0,877 | 2,530 | 0,143 |
| **Length of PrEP use in days** |  |  |  |  |
| <90 | Ref. |  |  |  |
| ≥90 - <180 | 7,552 | 4,679 | 12,668 | 0,000 |
| ≥180 - <365 | 19,110 | 12,348 | 31,039 | 0,000 |
| ≥365 - <545 | 40,421 | 25,790 | 66,372 | 0,000 |
| ≥545 - <730 | 52,096 | 32,772 | 86,603 | 0,000 |
| ≥730 - <1095 | 56,214 | 36,220 | 91,578 | 0,000 |
| ≥1095 | 53,030 | 34,650 | 85,401 | 0,000 |

**Chi-square test**

**Individuals' total PDC and age**

X-squared = 3.3235, df = 5, p-value = 0.6502

**Individuals' total PDC and place of residence**

X-squared = 16.093, df = 4, p-value = 0.002897

**Individuals' total PDC and length of PrEP use**

X-squared = 1063.2, df = 6, p-value < 2.2e-16
